# HEALTHY LIFESTYLE BEHAVIORS AND BIOLOGICAL AGING IN THE U.S. NATIONAL HEALTH AND NUTRITION EXAMINATION SURVEYS 1999-2018

**DOI:** 10.1093/geroni/igae098.0567

**Published:** 2024-12-31

**Authors:** Yian Gu

**Affiliations:** Columbia University, New York, New York, United States

## Abstract

TBD

